# Elucidation of the Mechanism of Action of Ginseng Against Acute Lung Injury/Acute Respiratory Distress Syndrome by a Network Pharmacology-Based Strategy

**DOI:** 10.3389/fphar.2020.611794

**Published:** 2021-01-20

**Authors:** Qi Ding, Wenxiang Zhu, Yirui Diao, Gonghao Xu, Lu Wang, Sihao Qu, Yuanyuan Shi

**Affiliations:** ^1^ School of Life Science, Beijing University of Chinese Medicine, Beijing, China; ^2^ Shenzhen Research Institute, Beijing University of Chinese Medicine, Shenzhen, China

**Keywords:** ginseng, ALI/ARDS, network pharmacology, molecular docking, PI3K-Akt signaling pathway, MAPK signaling pathway

## Abstract

Acute respiratory distress syndrome (ARDS) is a complex cascade that develops from acute lung injury (ALI). Ginseng can be used to treat ALI/ARDS. Studies have shown that some of ingredients in ginseng had anti-inflammation, antioxidative, and immune regulation effects and can protect alveolar epithelial cells in mice. However, the potential targets, biological processes, and pathways related to ginseng against ALI/ARDS have not been investigated systematically. We employed network pharmacology, molecular docking, and animal experiments to explore the therapeutic effects and underlying mechanism of action of ginseng against ALI/ARDS. We identified 25 compounds using ultrahigh-performance liquid chromatography Q-Orbitrap mass spectrometry and their 410 putative targets through database analyses. Sixty-nine of them were considered to be key targets of ginseng against ALI/ARDS according to overlapping with ALI/ARDS-related targets and further screening in a protein–protein interaction (PPI) network. The phosphatidylinositol 3-kinase-protein kinase B (PI3K-AkT) and mitogen-activated protein kinase (MAPK) pathways were recognized to have critical roles for ginseng in ALI/ARDS treatment. Signal transducer and activator of transcription (STAT) 3, vascular endothelial growth factor A (VEGFA), fibroblast growth factor (FGF) 2, phosphatidylinositol-4,5-bisphosphate 3-kinase catalytic subunit alpha (PIK3CA), MAPK1, and interleukin (IL) 2 were the top six nodes identified by analyses of a compound–target-pathway network. Molecular docking showed that most of the ingredients in ginseng could combine well with the six nodes. Ginseng could reduce the pathologic damage, neutrophil aggregation, proinflammatory factors, and pulmonary edema in vivo and inhibit the PI3K-Akt signaling pathway and MAPK signaling pathway through downregulating expressions of STAT3, VEGFA, FGF2, PIK3CA, MAPK1, and IL2. Our study provides a theoretical basis for ginseng treatment of ALI/ARDS.

## Introduction

Acute respiratory distress syndrome (ARDS) is a complex process which develops from acute lung injury (ALI). ARDS is characterized by acute and progressively increased dyspnea, refractory hypoxemia, and pulmonary edema (Wheeler and Bernard, 2007; Thompson et al., 2017). ALI/ARDS can be caused by pneumonia, gastric aspiration, viral/bacterial infection, or severe sepsis. (Sweeney and McAuley, 2016). In China, ∼20% of coronavirus disease 2019 (COVID-19) patients have the severe disease form of disease, which can develop rapidly into ALI/ARDS (Wu and McGoogan, 2020). ALI/ARDS is one of the most common diseases that seriously threatens the lives of patients (Sweeney and McAuley, 2016). ALI/ARDS caused by different risk factors has a common pathophysiological basis: excessive activation of immune cells, “cytokine storm,” oxidative stress, inflammation, hypoxia, and electrolyte disturbances (Sapru et al., 2015). Besides supportive therapy, anti-inflammatory, antioxidative, and anticoagulant agents, surfactants, and neuromuscular blockers have been used to treat ALI/ARDS (Fan et al., 2018). However, none of these treatments have been approved by the US Food and Drug Administration, European Medicines Agency, or the Chinese National Medical Products Administration because of weak efficacy or serious side effects.

Traditional Chinese medicine (TCM) has been used to treat various diseases in East Asia for thousands of years. According to clinical manifestations of ALI/ARDS, it can be classified as “Bao Chuan” or “Chuan Tuo.” “Qi” in the lungs cannot sink, and lung failure is the pathologic basis of ALI/ARDS.

*Panax ginseng* C. A. Mey (ginseng) is used widely worldwide (Lee et al., 2019). Ginseng can tonify lung Qi and is applied often to treat various respiratory diseases (Mancuso and Santangelo, 2017). The main active components of ginseng are ginsenosides, which have been shown to have varieties of beneficial effects (Mancuso and Santangelo, 2017). However, studies on ginseng or ginsenosides have focused mainly on the mechanism of a single target-oriented pathway or inflammatory regulation. This approach cannot fully explain the overall therapeutic effects and mechanism of action (MoA) of ginseng for ALI/ARDS treatment (Cheng and Li, 2016; Lee et al., 2018; Rajput et al., 2019). Thus, there is a need to investigate the MoA of ginseng for treating ALI/ARDS using advanced approaches.

TCM formulations contain multiple ingredients, targets, and pathways, which cannot be elucidated using traditional methods (Ma et al., 2016). Network pharmacology is based on analyses of big data and systems biology to integrate information on multiple compounds. Network pharmacology is becoming a promising tool to reveal the MoA of multiple-component drugs (Hopkins, 2008; Kibble et al., 2015). The concept of network pharmacology coincides closely with TCM concepts for treating diseases (Hao and Xiao, 2014). Increasing numbers of researchers have adopted network pharmacology to study the MoA of TCM components in treatment of diseases (Zhao et al., 2018; Guo et al., 2019; Wang et al., 2020).

**FIGURE 1:**
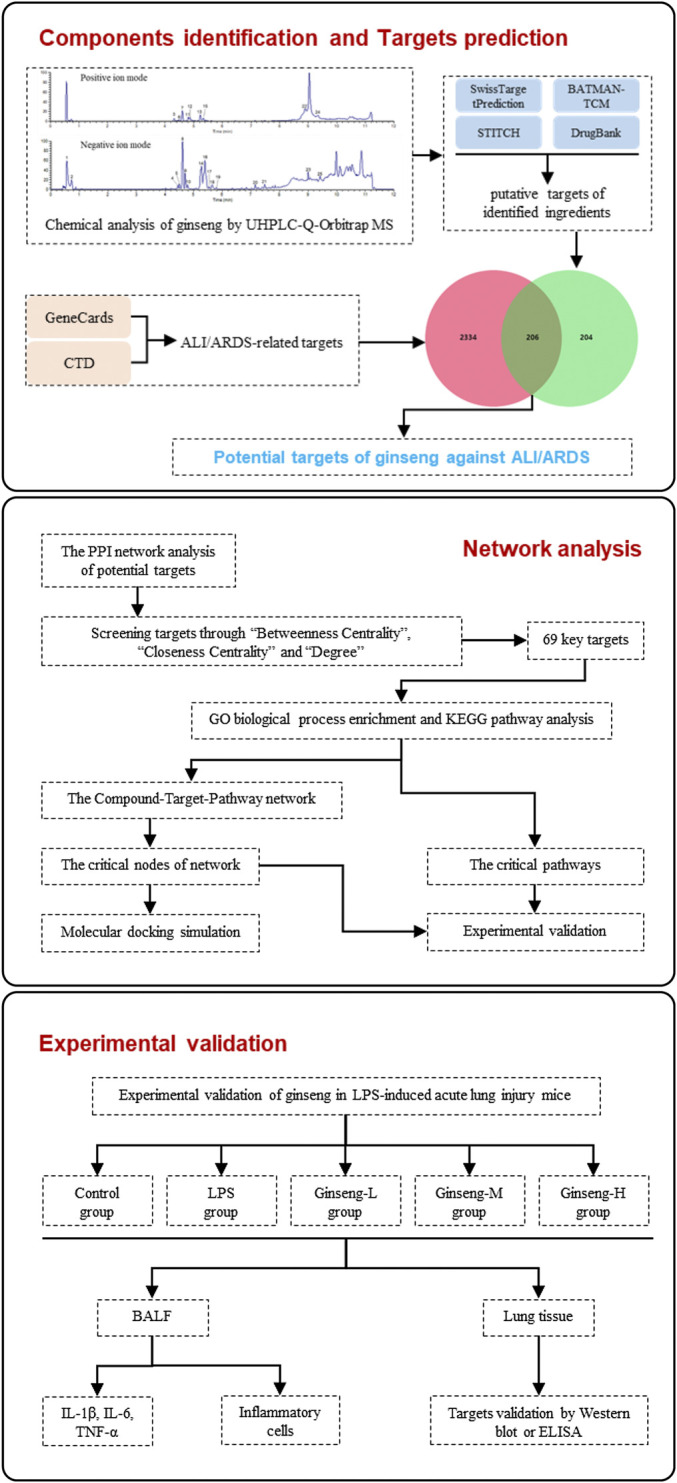
Procedures of a network pharmacology-based strategy for elucidating the pharmacologic mechanism of action of ginseng against ALI/ARDS.

Here, we first analyzed the chemical constituents of ginseng using ultrahigh-performance liquid chromatography Q-Orbitrap mass spectrometry (UHPLC-Q-Orbitrap MS). Then, network pharmacology was employed to identify putative targets, candidate pathways, and the therapeutic MoA of ginseng against ALI/ARDS. Subsequently, molecular docking was employed to verify the prediction results of network pharmacology. Furthermore, we validated the proposed pharmacologic MoA of ginseng on lipopolysaccharide- (LPS-) induced ALI/ARDS in a mouse model. Figure 1 shows the procedures of our study.

## Materials and Methods

### Preparation of Ginseng Sample

Ginseng was purchased from Tongrentang (Beijing, China) and identified by Doctor Ding Qi as the roots and rhizomes of *Panax ginseng* C. A. Mey. Sliced ginseng was decocted twice with purified water for 1 h each time. The combined extract was concentrated and freeze-dried to obtain lyophilized powder. The latter was stored at −80°C and used for mass detection and experimental validation.

### UHPLC and MS Conditions

The chemical constituents of ginseng were analyzed on a Thermo Dionex Ultimate 3000 UPLC system equipped with a binary pump, degasser, autosampler, and a column compartment (Thermo Fisher, Waltham, MA, United States). The sample was separated on a UHPLC HSS T3 column (2.1 × 100 mm, 1.8 μm; Waters, Milford, MA, United States) eluted with a mixture of 0.1% formic acid (A) and acetonitrile (B). The gradient was 0-1 min, maintained at 10% B; 1–8 min, increased linearly to 80% B; 8–11 min, increased to 90% B, and maintained at 90% B for 1 min. The flow rate was 0.5 ml/min, and the column temperature was set at 30°C.

Mass detection was undertaken on a Thermo Q-Exactive Orbitrap Mass Spectrometer (Thermo Fisher) equipped with an electrospray ionization source in positive and negative ion modes. Mass conditions were set as follows: capillary temperature, 320°C; spray voltage, 3.8 kV for positive and 3.1 kV for negative ion modes; sheath gas (N_2_) flow rate, 45 Arb; and collision energy, 40 eV. The full-scan mass spectrum was recorded in m/z 70–1,000 at seven spectra/s. MS/MS experiments were set as data-dependent scans. All data acquisitions were controlled by Thermo Xcalibur 4.0.27.

### Prediction of the Putative Targets of the Identified Constituents in Ginseng

The putative targets of the identified constituents of ginseng were obtained mainly from SwissTargetPrediction (www.swisstargetprediction.ch/) (Daina et al., 2019), BATMAN-TCM (www.bionet.ncpsb.org/batman-tcm/) (Liu et al., 2016), STITCH v5.0 (www.stitch.embl.de/) (Szklarczyk et al., 2016), and DrugBank v5.1.7 (www.drugbank.ca/) (Wishart et al., 2018). Then, UniProt (www.uniprot.org/) was used to convert the protein name of bioactive ingredients to the gene names (UniProt Consortium, 2019).

### Acquisition of ALI/ARDS-Related Targets

The known ALI/ARDS-related targets were obtained mainly from two databases using “acute lung injury” or “acute respiratory distress syndrome” as the keywords. One was the GeneCards database v5.0 (www.genecards.org/). GeneCards database is a searchable, integrative database that provides comprehensive, user-friendly information on all annotated and predicted human genes (Stelzer et al., 2016). The other source was the Comparative Toxicogenomics Database (CTD; www.ctdbase.org/), which provides manually curated information about chemical–gene/protein interactions, chemical–disease, and gene–disease relationships (Davis et al., 2019).

### Construction of a Protein–Protein Interaction (PPI) Network

Drug targets and ALI/ARDS-associated targets were intersected to obtain a Venn diagram of the intersected gene symbols. Then, the PPI network was created using the Search Tool for the Retrieval of Interacting Genes/Proteins (STRING) database v11.0 (www.string-db.org/) (Szklarczyk et al., 2019). Then, the result of the PPI network was saved and inputted into Cytoscape v3.7.2 (www.cytoscape.org/) for further analyses (Shannon et al., 2003). The parameters “Betweenness Centrality,” “Closeness Centrality,” and “Degree” were calculated to assess the topological importance of the nodes in the PPI network.

### Analyses of Pathway Enrichment

The Database for Annotation, Visualization and Integrated Discovery (DAVID) v6.8 (www.david.ncifcrf.gov/) provides a comprehensive set of functional annotation tools for investigators to understand biological meaning behind large lists of genes (Huang et al., 2009a; Huang et al., 2009b). DAVID was employed to carry out pathway enrichment analyses using the Gene Ontology (GO) and Kyoto Encyclopedia of Genes and Genomes (KEGG) databases. Pathway terms from these databases with *p* < 0.05 were regarded as significant.

### Simulation by Molecular Docking

The three-dimensional structures of small molecule compounds were obtained using the PubChem database and Chem3D software. The conformation of proteins was collected from the Protein Data Bank (PDB) database (www.rcsb.org/) (Burley et al., 2019). Then, the compounds and proteins were saved in Protein Data Bank, Partial Charge (Q), and Atom Type (T) (PDBQT) format after removing water, adding hydrogen bonds, detecting the root, setting rotatable bonds, and computing the Gasteiger charge using MGLTools 1.5.6 (www.mgltools.scripps.edu/). Subsequently, the active pocket was determined according to the binding position of the protein and its inhibitor. Finally, molecular docking was performed using AutoDock Vina 1.1.2 (www.vina.scripps.edu/) (Trott and Olson, 2010). PyMol 2.3.2 (www.pymol.org/) was used to visualize the results of molecular docking.

### Experimental Validation

#### LPS-Induced Animal Model and Drug Treatment

Adult male C57BL/6J mice (18–22 g, 7 weeks) were purchased from Sibeifu Biotechnology (animal license: SXYK (Jing) 2019-0010) in Beijing, China. Mice were housed in a temperature (25°C) and humidity (60%) controlled environment under a 12-h light/dark cycle and had free access to food and water. Pentobarbital sodium was used as an anesthetic to minimize pain during all procedures. All mice experiments were undertaken in accordance with the Guide for the Care and Use of Laboratory Animals (US National Institutes of Health, Bethesda, MD, United States) and the related ethical regulations of Beijing University of Chinese Medicine (Beijing, China). Mice were acclimatized to their surroundings for 1 week before experimentation.

Weight-matched mice were divided randomly into seven groups. After fasting for 12 h, mice were administered (via the trachea) LPS (Sigma Aldrich, Saint Louis, MO, United States) or 0.9% NaCl (control). Mice were administered (p.o.) ginseng (0.1, 0.5, and 1 g/kg) or prednisone (7 mg/kg; positive drug) once a day for 1 week before intratracheal administration of LPS. Mice in the control group and LPS group received an equivalent volume of medium. Twenty-four hours after LPS administration, all mice were executed by injection of excess pentobarbital sodium. Immediately afterward, lung tissue and bronchoalveolar lavage fluid (BALF) were collected. Lung tissue was weighed to calculate the pulmonary index (lung weight/bodyweight (mg/g)). The left upper lung was fixed in 10% formalin over 24 h and embedded in paraffin for histology. The right lower lung was excised, weighed, and dried at 65°C for 72 h to obtain the dry weight. The wet weight:dry weight (WW:DW) ratio of the lung was calculated to evaluate edema in lung tissue. The remaining lung tissue was frozen rapidly and stored at −80°C.

#### Counts of Inflammatory Cells and Expression of Proinflammatory Cytokines in BALF

BALF samples were collected as described previously (Ju et al., 2018) and centrifuged at 1,000 × *g* for 5 min at 4°C. Precipitates (cell pellets) were resuspended in phosphate-buffered saline. The total cell count was determined using a hemocytometer. Then, cells were stained with Wright–Giemsa solution (Solarbio, Beijing, China) and classified based on nuclear morphology and color into neutrophils and macrophages.

#### Histopathology

Lung tissue sections (4–5 μm in thickness) were stained with hematoxylin and eosin (H&E). All sections were examined and assessed under light microscopy by very experienced pathologists. Inflammation was evaluated according to the degree of lung tissue lesions, infiltration of inflammatory cells, thickening of alveoli septa, and hyperplasia of fibrous connective tissue.

#### Enzyme-Linked Immunosorbent Assay (ELISA)

Expression of interleukin-1β (IL-1β), IL-6, tumor necrosis factor-α (TNF-α), IL-2, vascular endothelial growth factor A (VEGFA), and fibroblast growth factor (FGF) 2 in BALF or lung tissue was measured with ELISA kits according to manufacturer (Proteintech, Wuhan, China) protocols.

#### Western Blotting

Immunoblot analyses of lung tissue were performed on total lysates as described by Ding et al. (2019) using phospho-signal transducer and activator of transcription (STAT) 3 (p-STAT3; catalog number, ab76315; Abcam, Cambridge, United Kingdom), STAT3 (ab68153; Abcam), phosphatidylinositol-4,5-bisphosphate 3-kinase catalytic subunit alpha (PIK3CA; ab40776; Abcam), phospho-mitogen-activated protein kinase (MAPK) 1 (p-MAPK1; ab201015; Abcam), MAPK1 (ab184699; Abcam), and glyceraldehyde 3-phosphate dehydrogenase (GAPDH; 60004-1-Ig, Proteintech) as primary antibodies and then incubation with the corresponding secondary antibodies. Protein bands were detected by an electrochemiluminescence reagent (NCM Biotech, Beijing, China). The intensity of protein bands was analyzed using Image-Pro Plus (Media Cybernetics, Rockville, ML, United States) and presented as the ratio to GAPDH.

#### Statistical Analyses

Data are the mean ± SD from at least three independent experiments. Statistical analyses of data were undertaken by one-way ANOVA followed by Student’s two-tailed *t*-test using Prism 8 (GraphPad, San Diego, CA, United States). *p* < 0.05 was considered significant.

## Results

### Identification of the Chemical Constituents of Ginseng

**FIGURE 2:**
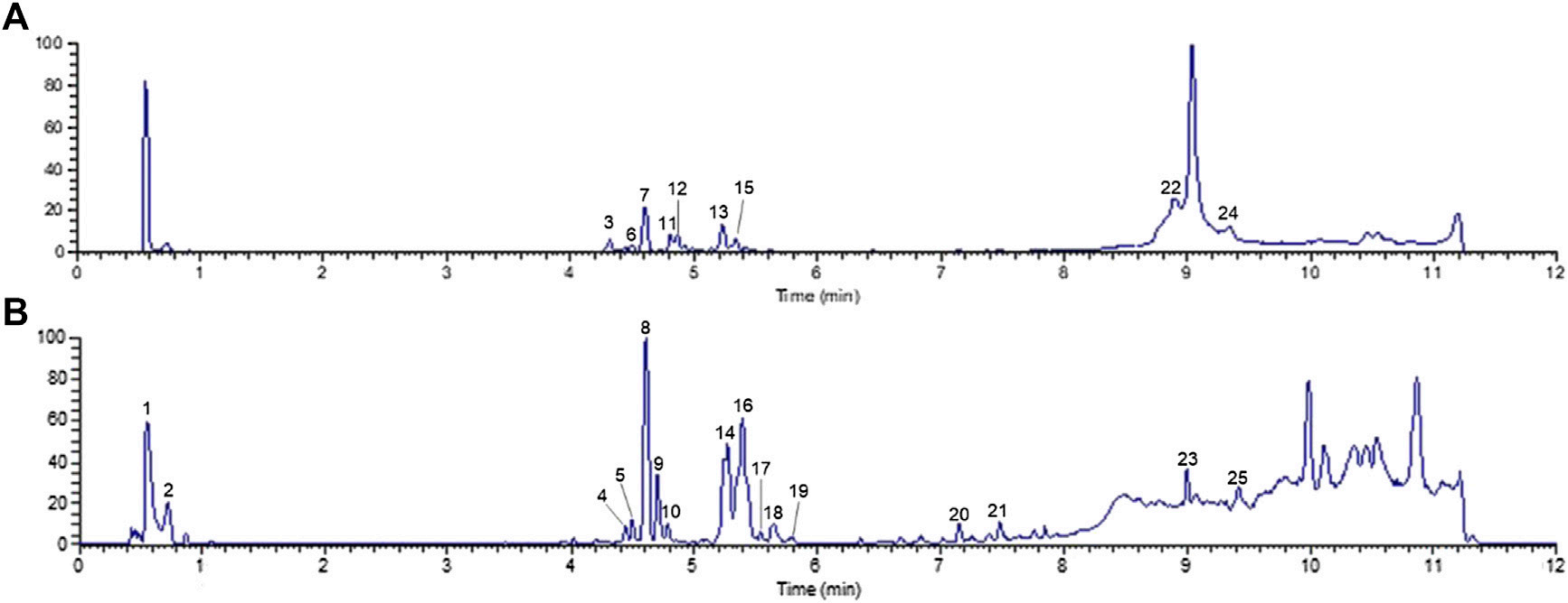
Total ion chromatogram monitored in positive **(A)** and negative **(B)** ion modes for ginseng.

UHPLC-Q-Orbitrap MS was applied to analyze the chemical constituents in ginseng. The major components were well separated and detected under optimized UHPLC and MS conditions (Figure 2). Twenty-five constituents in ginseng (e.g., ginsenosides, organic acids, and ginsenoyne) (Table 1) were identified by comparison with the literatures (Xu et al., 2017; Zhou et al., 2018; Shi et al., 2020) according to accurate mass, chromatographic behavior, and mass of fragments ions.

**TABLE 1:** Identified constituents of ginseng by UHPLC-Q-Orbitrap MS.

No.	RT (min)	ESI-MS	Molecular formula	Error (ppm)	Compound	PubChem CID
1	0.56	195.04988 [M-H]^−^	C_6_H_12_O_7_	−3.179	Galactonic acid	128869
2	0.73	115.00293 [M-H]^−^	C_4_H_4_O_4_	−1.478	Maleic acid	444266
3	4.32	843.51091 [M+H]^+^	C_44_H_74_O_15_	0.368	Vina ginsenoside R1	44584744
4	4.44	961.53759 [M-H]^−^	C_48_H_82_O_19_	0.406	Ginsenoside Rd	24721561
5	4.49	765.47824 [M-H]^−^	C_42_H_70_O_12_	−0.862	Ginsenoside Rg6	91895489
6	4.50	979.54844 [M+H]^+^	C_48_H_82_O_20_	0.653	Ginsenoside I	102050355
7	4.61	947.55818 [M+H]^+^	C_48_H_82_O_18_	0.295	Ginsenoside Re	441921
8	4.62	799.48492 [M-H]^−^	C_42_H_72_O_14_	0.650	Ginsenoside Rg1	441923
9	4.70	769.47438 [M-H]^−^	C_41_H_70_O_13_	0.754	Ginsenoside F3	46887678
10	4.78	619.42047 [M-H]^−^	C_36_H_60_O_8_	−0.856	Ginsenoside Rk3	75412555
11	4.82	957.50577 [M+H]^+^	C_48_H_76_O_19_	−0.136	Ginsenoside Ro	11815492
12	4.87	1211.64320 [M+H]^+^	C_58_H_98_O_26_	0.578	Ginsenoside Ra1	100941542
13	5.23	801.50052 [M+H]^+^	C_42_H_72_O_14_	0.649	Ginsenoside Rf	441922
14	5.27	1107.59562 [M-H]^−^	C_54_H_92_O_23_	0.469	Ginsenoside Rb1	9898279
15	5.34	1079.60062 [M+H]^+^	C_53_H_90_O_22_	0.389	Ginsenoside Rb2	6917976
16	5.39	1077.58492 [M-H]^−^	C_53_H_90_O_22_	0.390	Ginsenoside Rb3	12912363
17	5.53	783.49018 [M-H]^−^	C_42_H_72_O_13_	0.868	Ginsenoside Rg2	21599924
18	5.65	783.49018 [M-H]^−^	C_42_H_72_O_13_	0.868	Ginsenoside Rg3	9918693
19	5.79	945.54311 [M-H]^−^	C_48_H_82_O_18_	0.857	Ginsenoside Rd1	102221467
20	7.15	621.43704 [M-H]^−^	C_36_H_62_O_8_	0.708	Ginsenoside Rh2	119307
21	7.48	619.42196 [M-H]^−^	C_36_H_60_O_8_	1.550	Ginsenoside Rh4	21599928
22	8.89	477.39350 [M+H]^+^	C_30_H_52_O_4_	−1.885	Panaxatriol	73599
23	9.00	459.38340 [M-H]^−^	C_30_H_52_O_3_	-0.871	Panaxadiol	73498
24	9.34	277.17990 [M+H]^+^	C_17_H_24_O_3_	−1.804	Ginsenoyne C	5317634
25	9.52	455.35300 [M-H]^−^	C_30_H_48_O_3_	1.098	16-Oxoseratenediol	5320337

### Putative Targets of Ginseng Constituents

Based on SwissTargetPrediction, BATMAN-TCM, STITCH, and DrugBank databases, 410 putative targets of ginseng were obtained after deletion of redundant items. Detailed information about which putative targets interacted with the 25 identified compounds is provided in Supplementary Data Sheet S1.

### Acquisition of Known ALI/ARDS-Related Targets

**FIGURE 3:**
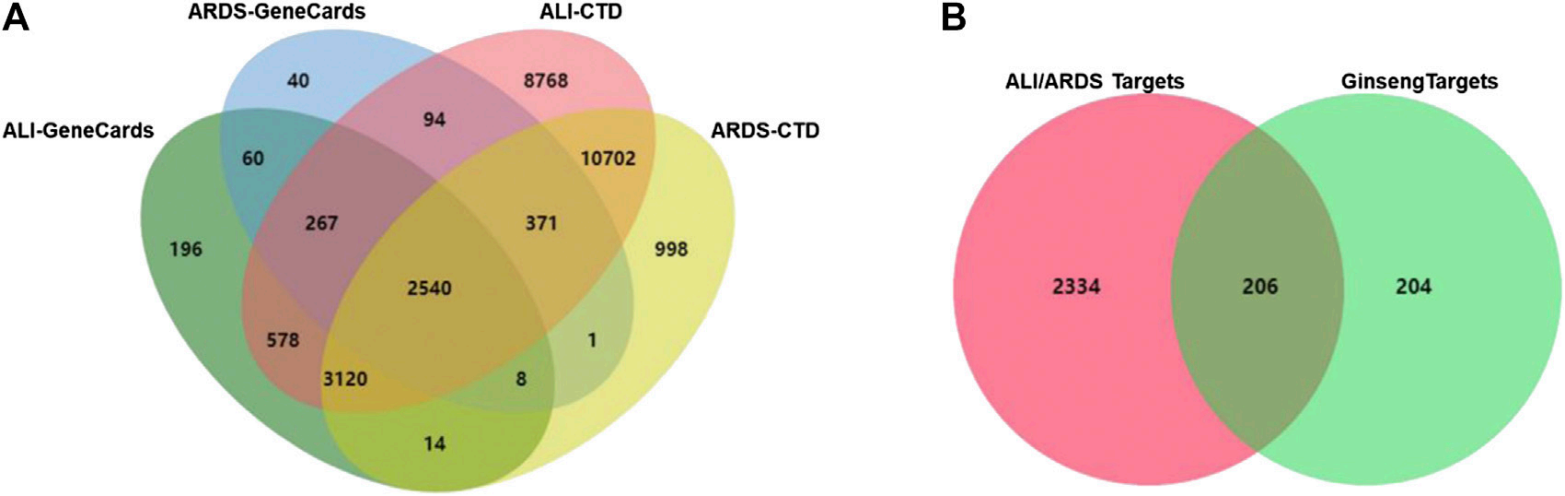
Venn diagram. **(A)** Venn diagram of candidate targets between ALI- and ARDS-associated targets from GeneCards and CTD databases. **(B)** Venn diagram of potential targets in ALI/ARDS and ginseng.

A total of 3,381 targets for ARDS and 6,783 targets for ALI were collected from the GeneCards database. A total of 17,754 targets for ARDS and 26,440 targets for ALI were obtained from the CTD database. As shown in Figure 3A, the 2,540 overlaps were considered to be ALI/ARDS-related targets. To obtain the targets of ginseng against ALI/ARDS, 410 targets from the identified components of ginseng were combined with 2,540 ALI/ARDS-related targets by using a Venn diagram. Finally, 206 targets were obtained and could be the potential targets for ALI/ARDS treatment by ginseng (Figure 3B). Detailed information on the 206 potential targets is provided in Supplementary Data Sheet S2.

### Analyses of the PPI Network

**FIGURE 4:**
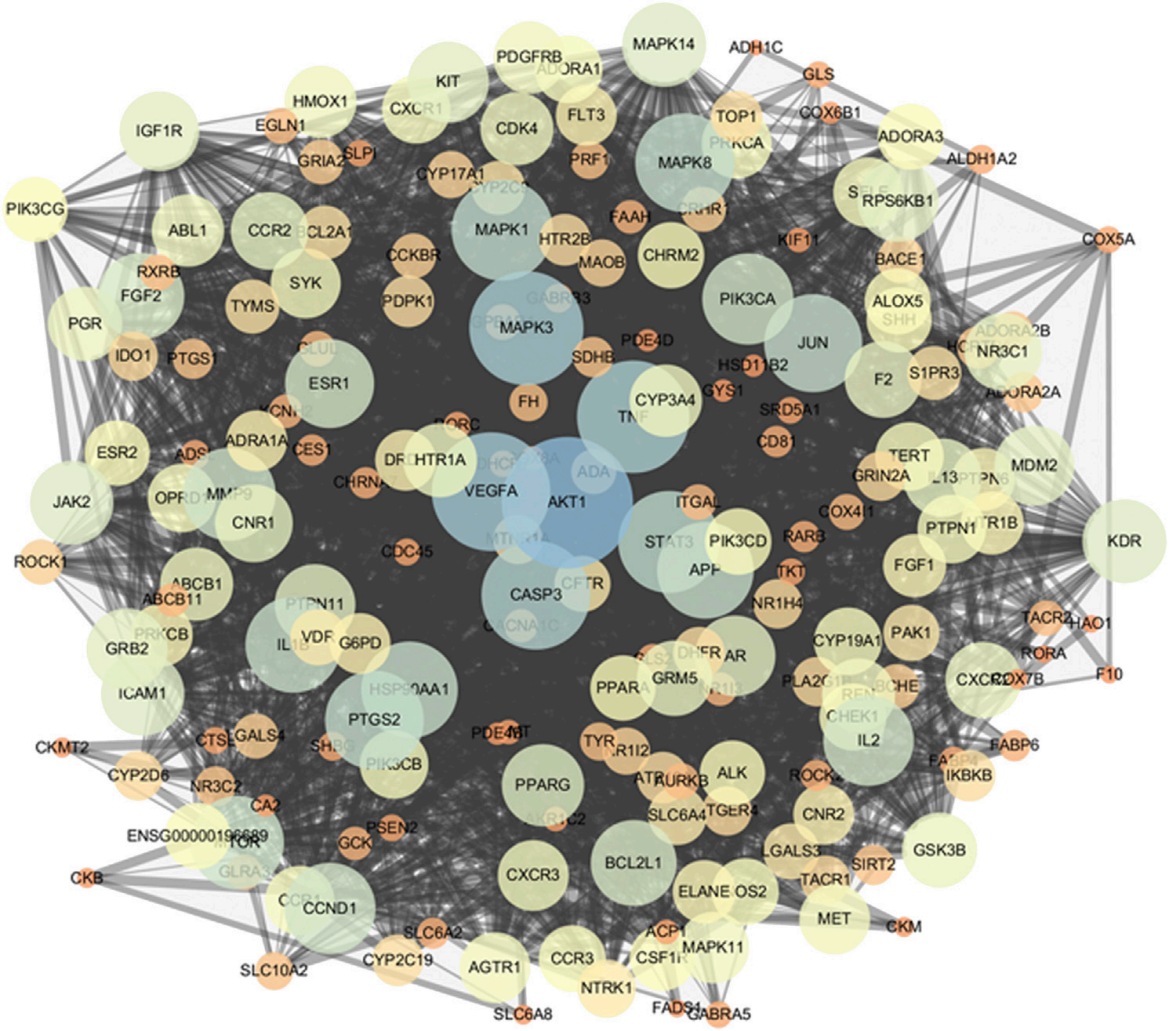
PPI network of potential targets. As the degree of targets increased, the circle became bigger and the color changed from orange to blue. As the combined score increased, the combined line became thicker.

The PPI network was constructed in the STRING 11.0 database to explore the underlying interactions of the 206 potential targets. The minimum combined score between targets was set as the medium confidence (0.400). Subsequently, the PPI network of potential targets was inputted into Cytoscape 3.7.2 for visualization. The PPI network consisted of 206 nodes and 2,490 edges (Figure 4). To identify targets which may have critical roles in the entire PPI network, three topological features (betweenness centrality, closeness centrality, and degree) of the nodes were calculated. Sixty-nine nodes were identified as the key targets of ginseng against ALI/ARDS because their three topological features were greater than the corresponding median values (betweenness centrality > 0.00249, closeness centrality > 0.4622, and degree > 18) (Supplementary Data Sheet S3).

### Analyses of Pathway Enrichment Using GO and KEGG Databases

**FIGURE 5:**
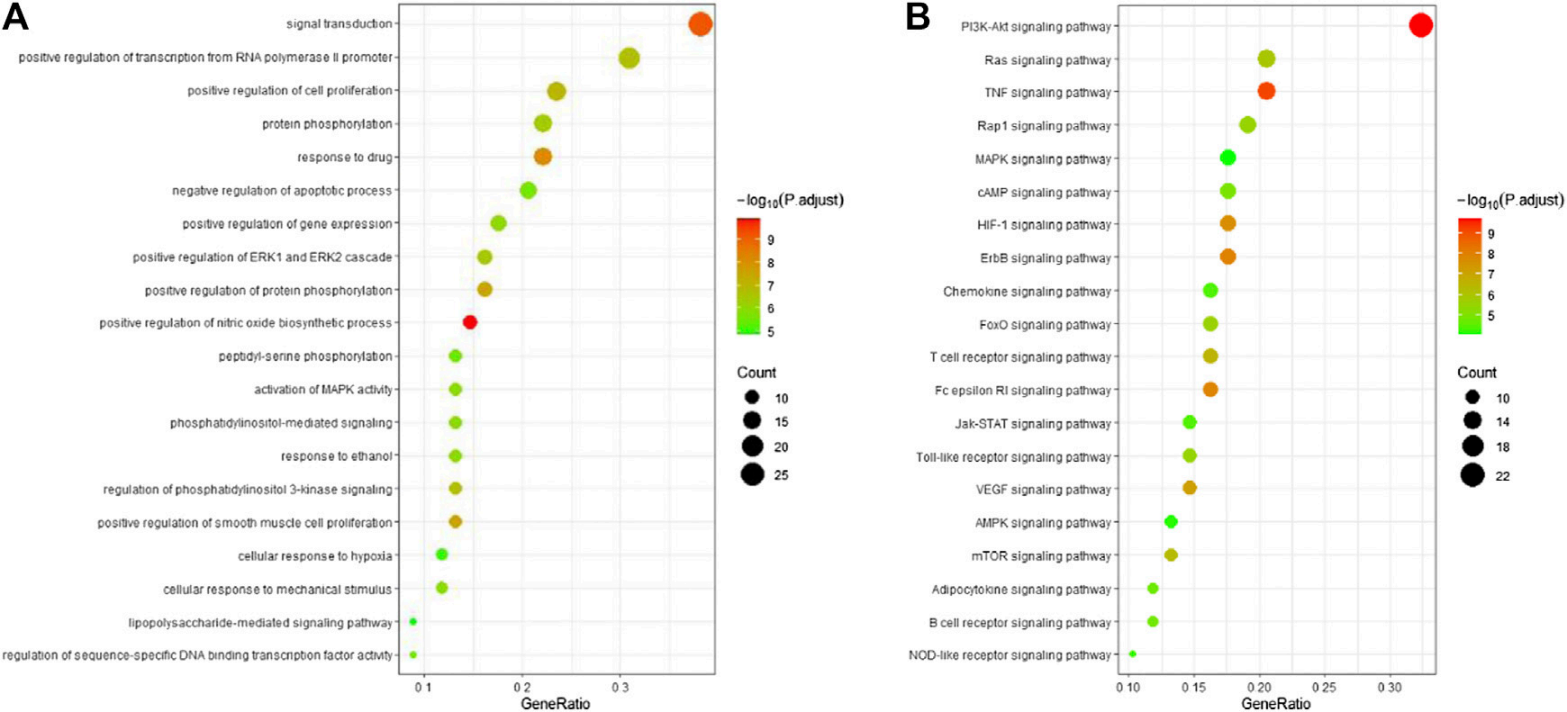
Bubble diagram of functional analysis. **(A)** Analyses of the 69 key targets using the GO database. **(B)** Pathway enrichment analyses of 69 key targets using the KEGG database. Adjusted *p* < 0.01.

To investigate the biological functions and anti-ALI/ARDS mechanisms of ginseng, the 69 key targets identified were selected for pathway enrichment analyses using GO and KEGG databases by DAVID Bioinformatics Resources 6.8. For a brief demonstration, only the top 20 significant (adjusted *p* < 0.01) GO entries were chosen for further analyses (Figure 5A). Results indicated that multiple biological processes were involved in ALI/ARDS treatment, including signal transduction, protein phosphorylation, positive regulation of the biosynthetic process of nitric oxide, regulation of phosphatidylinositol 3-kinase (PI3K) signaling, and activation of MAPK activity.

Pathway enrichment analyses using the KEGG database were undertaken to investigate the corresponding pathologic processes of ginseng key targets in ALI/ARDS treatment. The diseases were caused by dysfunctions of basic biology, so the KEGG-pathway sections of human diseases were removed (Xu et al., 2018). After screening by a parameter-adjusted *p* < 0.01, the top 20 significant pathway terms using the KEGG database were obtained, and a “bubble diagram” was constructed (Figure 5B). According to the pathogenesis of ALI/ARDS, the KEGG pathway terms could be divided into inflammation (e.g., TNF signaling pathway and hypoxia inducible factor- (HIF-) 1 signaling pathway), immune response (e.g., T cell receptor signaling pathway and B cell receptor signaling pathway), energy metabolism (AMPK signaling pathway), and signal transduction (e.g., PI3K-protein kinase B (Akt) signaling pathway, Ras signaling pathway, and MAPK signaling pathway). Based on these results, the PI3K-Akt signaling pathway and MAPK signaling pathway were highly enriched in the GO and KEGG databases. These results indicated that ginseng worked mainly through the PI3K-Akt signaling pathway and MAPK signaling pathway in ALI/ARDS treatment.

### Analyses of the Compound–Target-Pathway Network

**FIGURE 6:**
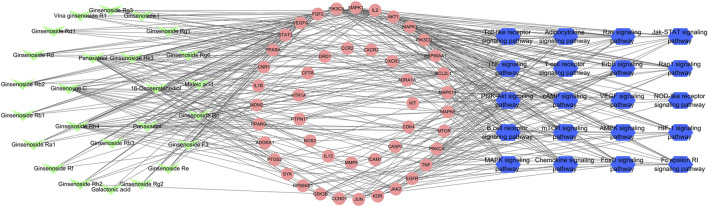
Compound–target-pathway network of identified compounds, key targets, and top 20 pathways using the KEGG database. Green “V” nodes represent the compounds in ginseng, red circle nodes represent the key targets, and blue hexagons represent the pathways enriched according to the KEGG database.

According to the predicted results of the identified compounds, key targets, and top 20 related pathways using the KEGG database, an integrated compound–target-pathway network was constructed using Cytoscape. The compound–target-pathway network consisted of 91 nodes and 411 edges (Figure 6). Nodes with higher betweenness centrality, closeness centrality, and degree as well as shorter average shortest path length in the compound–target-pathway network were considered to be vital. STAT3, VEGFA, FGF2, PIK3CA, MAPK1, and IL2 were the top six nodes (Supplementary Data Sheet S4). These nodes also interacted closely with most of the pathways (including the PI3K-Akt signaling pathway and MAPK signaling pathway) and compounds.

### Molecular Docking

**FIGURE 7:**
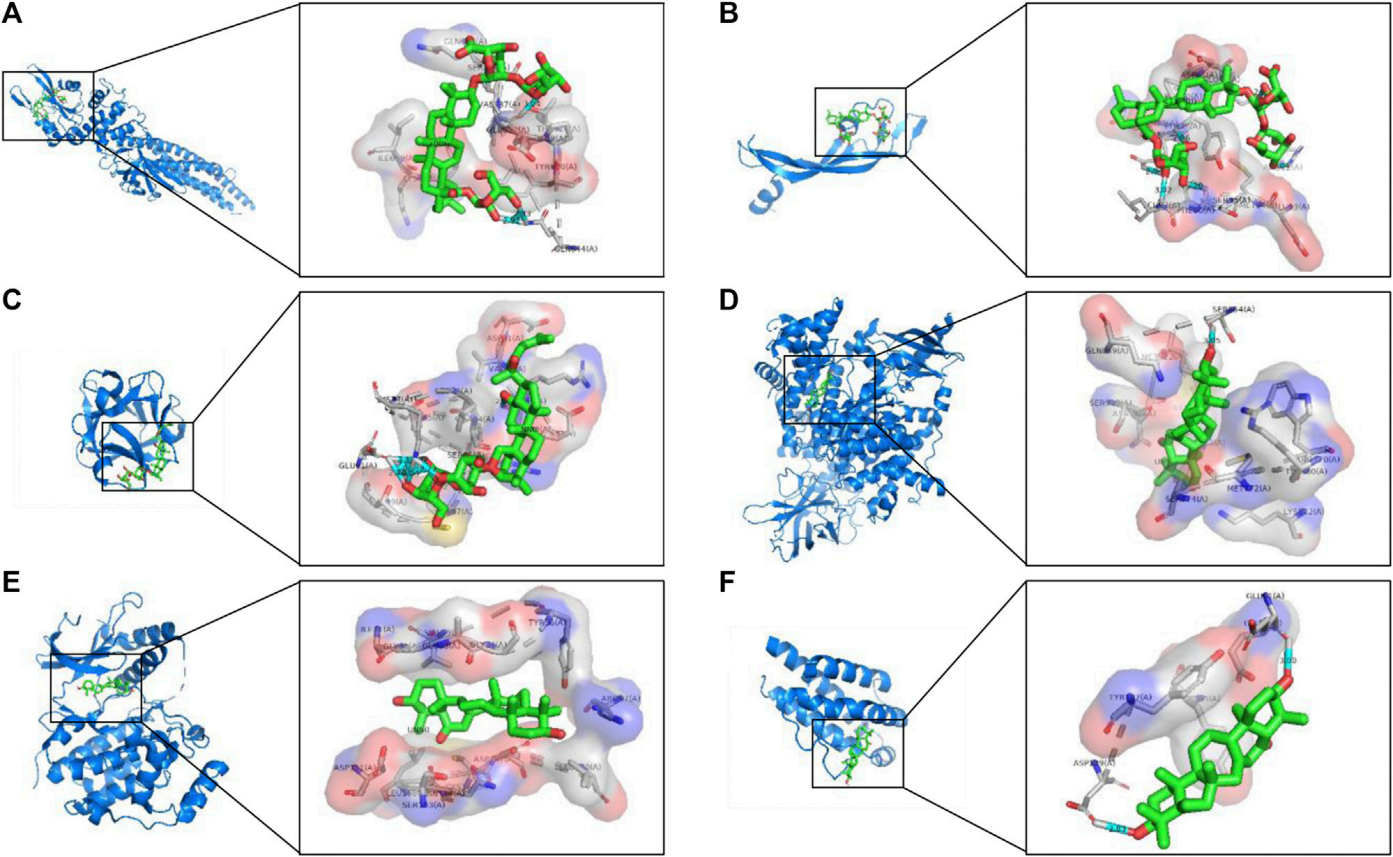
Analyses of target–compound docking simulation. **(A)** Ginsenoside Ro acts on STAT3. **(B)** Ginsenoside Ro acts on VEGFA. **(C)** Ginsenoside Rg3 acts on FGF2. **(D)** 16-Oxoseratenediol acts on PIK3CA. **(E)** 16-Oxoseratenediol acts on MAPK1. **(F)** 16-Oxoseratenediol acts on IL2. A ball-and-stick represents the compound, a blue chain represents the target, and hydrogen bonds are represented by a blue line.

The network pharmacology results revealed the potential key targets and pathways of ginseng against ALI/ARDS. To verify the reliability of the prediction results of network pharmacology, the 25 compounds were docked with STAT3, VEGFA, FGF2, PIK3CA, MAPK1, and IL2 using AutoDock Vina. The Vina score (affinity (kcal/mol)) indicates the binding activity between the receptor and ligand. The more negative the Vina score, the more stable is the ligand binding to the receptor (Liu et al., 2020). The Vina scores of the 25 identified compounds and top six targets are listed Supplementary Data Sheet S5. Most of the compounds had good binding activity with STAT3, VEGFA, FGF2, PIK3CA, MAPK1, and IL2. Using PyMOL, the top predicted target–compound pair in terms of affinity was employed as an example for visualization (Figure 7).

### Experimental Validation of Ginseng in a Model of LPS-Induced ALI in Mice

#### Ginseng Treatment Attenuated LPS-Induced ALI in Mice

According to the results of network pharmacology and molecular docking, we considered that the interaction between the active constituents in ginseng and key targets was the basis of their biological activity. Hence, ginseng had the characteristics of multiple ingredients, multiple targets, and multiple pathways in ALI/ARDS treatment. Therefore, the therapeutic effects and MoA of ginseng against ALI/ARDS in vivo were explored to validate the results of network pharmacology.

**FIGURE 8:**
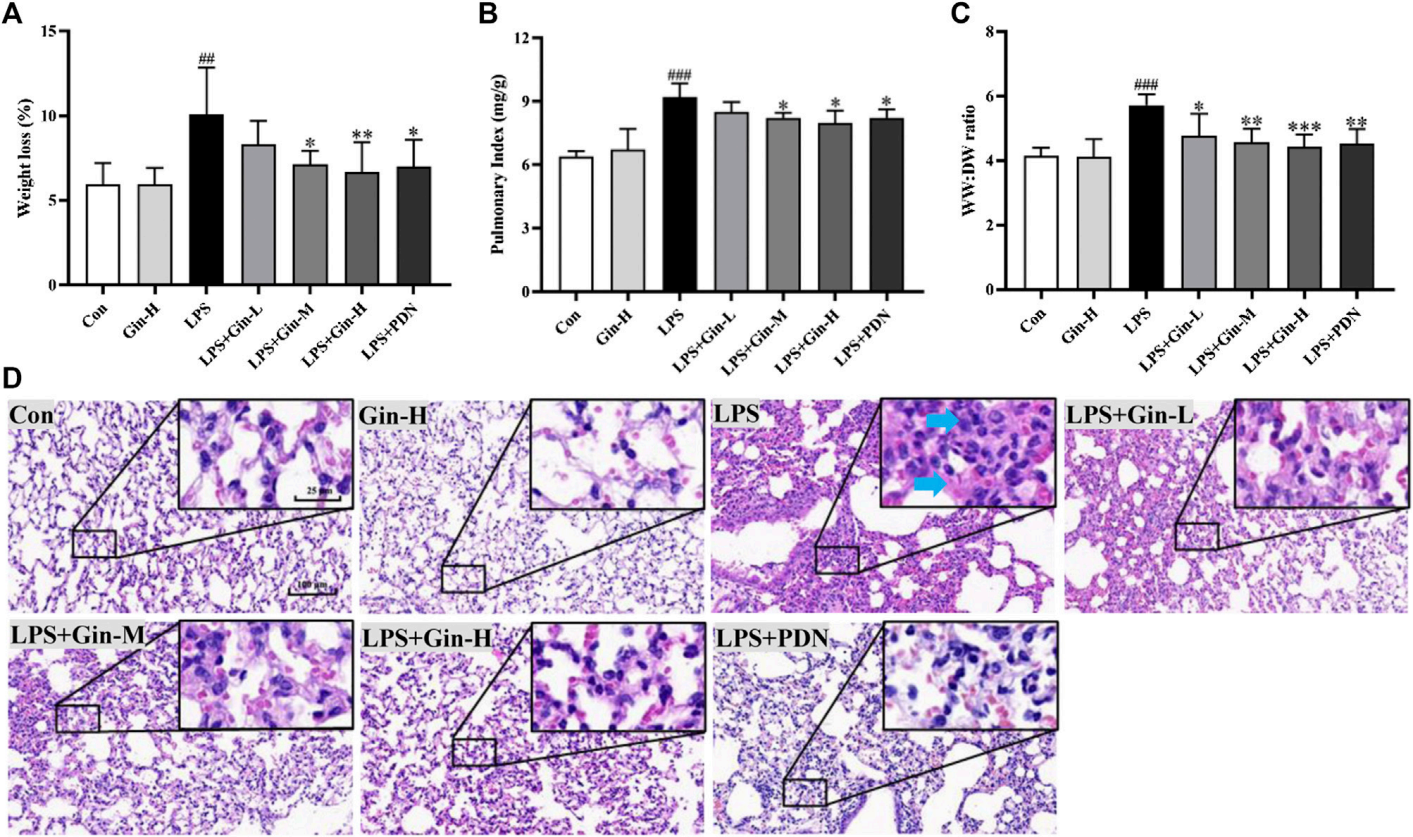
Ginseng treatment attenuates LPS-induced acute lung injury in mice. Mice were administered (p.o.) ginseng (Gin, 0.1, 0.5, and 1 g/kg) or prednisone (PDN, 7 mg/kg, positive drug) once a day for 1 week before intratracheal instillation of LPS **(A)** Weight loss, **(B)** pulmonary index, and **(C)** WW:DW ratio of the lung are displayed in histograms; **(D)** representative H&E-stained section of lung tissue (blue arrow shows inflammatory cell infiltration or alveolar wall thickening). Data are the mean ± SD (*n* = 7). ##*p* < 0.01 and ###*p* < 0.001 vs. the control group; *p < 0.05, **p < 0.01, and ***p < 0.001 vs. the LPS group.

First, we evaluated the effects of ginseng on LPS-induced ALI in mice. Compared with the control group, mice in the LPS group showed a significant increase in bodyweight loss, pulmonary index, and WW:DW ratio of the lung (Figures 8A–C). However, ginseng treatment could decrease the weight loss, pulmonary index, and WW:DW ratio of the lung in a dose-dependent manner. The histopathologic changes in lung tissues of mice were assessed by H&E staining (Figure 8D). Lung tissues were damaged severely by LPS, with obvious alveolar wall thickening and inflammatory cell infiltration (shown as blue arrows in Figure 8), and these injuries were alleviated after treatment with ginseng or prednisone. These results demonstrated that ginseng could inhibit LPS-induced ALI in mice efficaciously.

#### Ginseng Treatment Reduced the Number of Inflammatory Cells and Expression of Proinflammatory Cytokines in BALF

**FIGURE 9:**
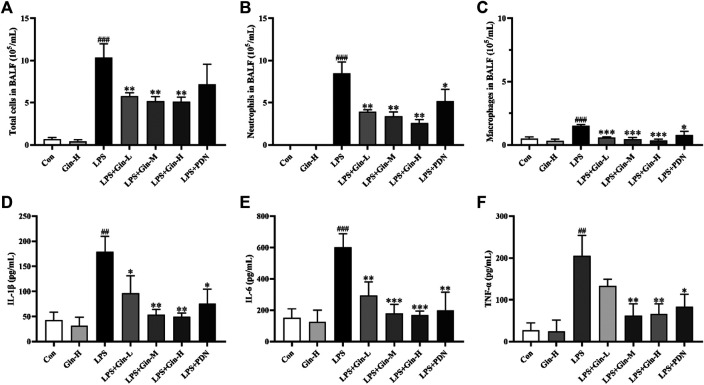
Ginseng treatment reduced the number of inflammatory cells and expression of proinflammatory cytokines in BALF. Mice were administered (p.o.) ginseng (Gin, 0.1, 0.5, and 1 g/kg) or prednisone (PDN, 7 mg/kg, positive drug) once a day for 1 week before intratracheal instillation of LPS **(A–C)** The number of total cells, neutrophils, and macrophages in BALF was determined by counting microscopic fields after staining with Wright–Giemsa solution. **(D–F)** Expressions of IL-1β, IL-6, and TNF-α in BALF were measured by ELISA. Data are the mean ± SD (*n* = 3). ^*##*^ *p* < 0.01 and ^*###*^ *p* < 0.001 vs. the control group; *p < 0.05, **p < 0.01, and ***p < 0.001 vs*.* the LPS group.

Network pharmacology indicated that the MoA of ginseng for ALI/ARDS involved several potential mechanisms, including inflammatory regulation and the immune response. Excessive inflammation and unbalanced immune homeostasis have been implicated in ALI/ARDS pathophysiology. To ascertain if ginseng regulates inflammation and the immune response in vivo, BALF was obtained to assess the alveolar microenvironment and inflammation. Compared with the control group, mice with LPS-induced ALI had a remarkably increased number of total cells, neutrophils, and macrophages in BALF (*p* < 0.001) (Figures 9A–C). In contrast, treatment with all doses of ginseng could reduce cell numbers significantly in BALF (*p* < 0.01). Prednisone treatment could also reduce the number of neutrophils significantly (*p* < 0.05), but had no significant effect on the total cell number. The effects of ginseng on proinflammatory cytokines in BALF are shown in Figures 9D–F. Compared with that in the control group, expression of IL-1β, IL-6, and TNF-α was increased markedly in the BALF of mice after induction by LPS, but obviously inhibited upon ginseng treatment compared with that in the LPS group. These results showed that ginseng could alleviate the inflammatory reaction in the alveoli of LPS-induced mice.

#### Validation of Targets and Pathways

**FIGURE 10:**
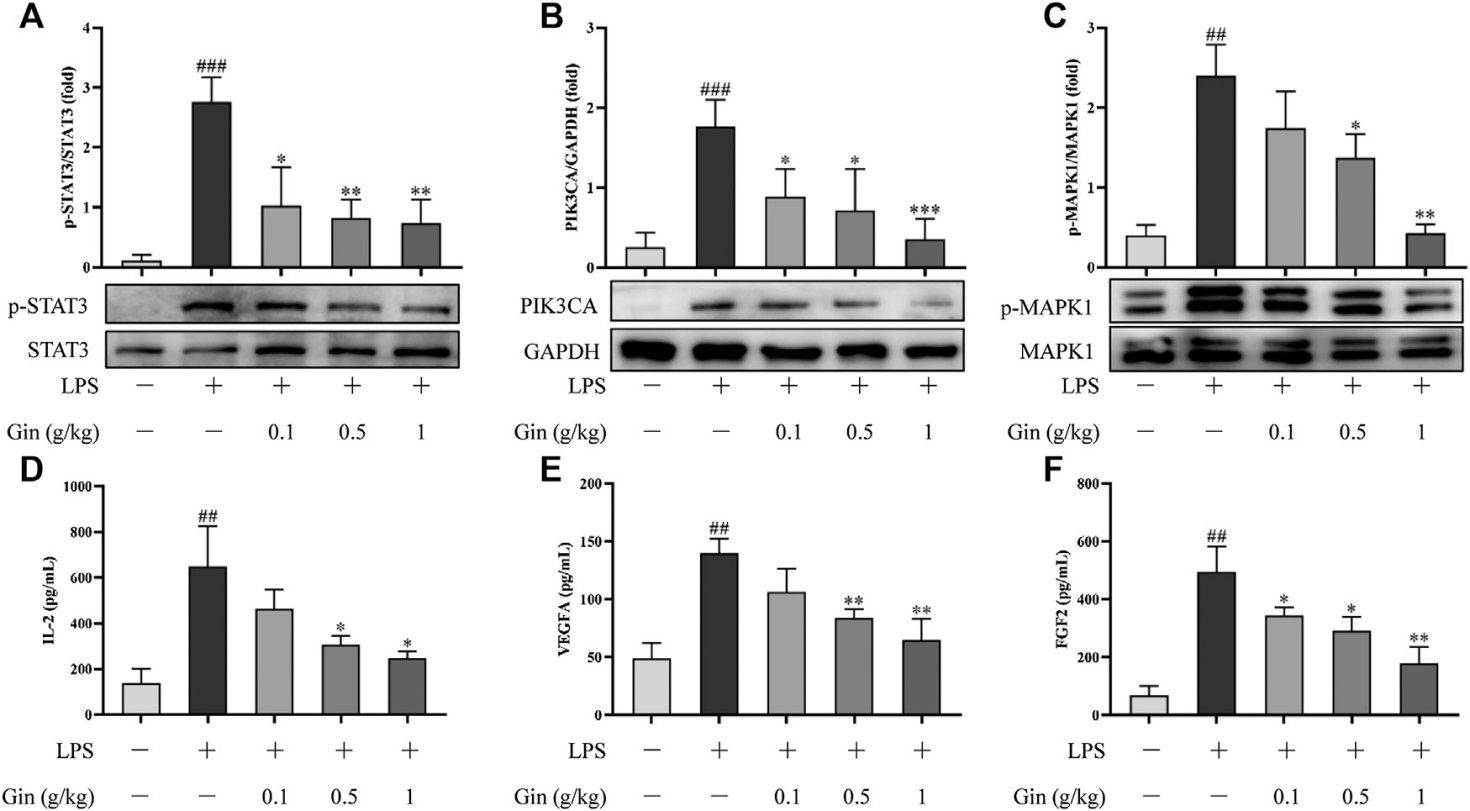
Ginseng treatment reduced the expression of STAT3, VEGFA, FGF2, PIK3CA, MAPK1, and IL2 in lung tissue after LPS-induced ALI in mice. (**A–C)** Expression of p-STAT3, PIK3CA, and *p*-MAPK1 in lung tissue was detected by Western blotting. **(D–F)** Expression of IL-2, VEGFA, and FGF2 in lung tissue was measured by ELISA. Data are the mean ± SD (*n* = 3). ^*##*^ *p* < 0.01 and ^*###*^ *p* < 0.001 vs. the control group; *p < 0.05, **p < 0.01, and ***p < 0.001 vs. the LPS group.

To verify the reliability of the prediction results of network pharmacology, expression of STAT3, VEGFA, FGF2, PIK3CA, MAPK1, and IL2 in ALI mice was measured by Western blotting or ELISA. Compared with that in the control group, expression of p-STAT3, VEGFA, FGF2, PIK3CA, p-MAPK1, and IL2 was increased significantly in the lung tissue of LPS-induced mice (*p* < 0.01) (Figure 10). Ginseng treatment could reduce the LPS-induced upregulation of expression of p-STAT3, VEGFA, FGF2, PIK3CA, p-MAPK1, and IL2 in a dose-dependent manner. VEGFA, FGF2, PIK3CA, MAPK1, and IL2 were the key targets involved in the PI3K-AkT signaling pathway. MAPK1 and FGF2 as well as IL1B and TNF (measured by ELISA) were the critical targets participating in the MAPK signaling pathway. These results suggested that ginseng inhibited the PI3K-AkT signaling pathway and MAPK signaling pathway mainly through these targets in LPS-induced ALI in mice.

## Discussion

ALI is a process in which a series of pathologic changes are caused by damage to lung tissue; often, ALI develops into ARDS (Thompson et al., 2017). It has been reported that ALI/ARDS pathogenesis is accompanied by disorders of lung immune homeostasis, organ inflammation, systemic inflammation, and apoptosis of functional cells, which lead to organ failure and endanger life (Sapru et al., 2015). ALI/ARDS is a refractory disease of the respiratory system. Corticosteroids, surfactants, *N*-acetylcysteine, statins, and beta-agonists are the agents prescribed to patients with ALI/ARDS. However, whether these agents help to reduce mortality in patients with ARDS, the duration of mechanical ventilation, or increase the number of ventilator-free days, is not known (Lewis et al., 2019). Therefore, it is necessary to discover novel and efficacious curative agents for ALI/ARDS patients.

Ginseng has multiple beneficial features and proven efficacy in treatment of acute respiratory illness in clinical and experimental research (Lee et al., 2012; Lee et al., 2018). However, the specific pharmacologic MoA of ginseng in ALI/ARDS treatment is incompletely understood.

We employed network pharmacology to explore the therapeutic MoA of ginseng on ALI/ARDS. First, the chemical profile of ginseng was mapped comprehensively by UHPLC-Q-Orbitrap MS, and 25 compounds were identified. Then, we revealed 206 potential targets of ginseng ingredients that have important roles in ALI/ARDS treatment, and 69 of them were identified as key targets in the PPI network. Pathway enrichment analyses using GO and KEGG databases suggested that the PI3K-AkT and MAPK signaling pathways were the principal pathways in ALI/ARDS treatment by ginseng.

PI3Ks are a large family of lipid enzymes that phosphorylate the 3′-OH group of phosphatidylinositol molecules at the plasma membrane (De Santis et al., 2019). Class-I PI3Ks are composed of a regulatory subunit and a catalytic subunit. Class-I PI3Ks have been demonstrated to be involved in cancer, immune disease, and inflammatory disease in humans (Fruman et al., 2017). Activated PI3K generates phosphatidylinositol (3,4,5)-trisphosphate. The latter is a second messenger that facilitates the translocation of Ser/Thr kinase AKT to the plasma membrane. Phosphorylated AkT has a pivotal role in important cellular functions because it phosphorylates various substrates. It has been reported that activation of the PI3K-Akt signaling pathway interacts with multiple pathways in ALI/ARDS. PI3K p110α is one of the catalytic subunits encoded by PIK3CA. PI3K p110α can be activated by the Toll-like receptor signaling pathway, Janus kinase- (Jak-) STAT signaling pathway, and B cell receptor signaling pathway. This activation induces the secretion of proinflammatory cytokines and recruitment of inflammatory cells into the lung. Moreover, upregulation of the PI3K-Akt signaling pathway promotes expression of IL2, FGF2, and VEGFA, which aggravates inflammation, induces epithelial cell apoptosis, and increases vascular permeability and pulmonary edema (Kelly et al., 2002; Yu et al., 2016; Cheng et al., 2020). Our in vivo experiments showed that ginseng could significantly reduce the pathologic damage, infiltration of proinflammatory factors, and pulmonary edema induced by LPS and downregulate the PI3K-Akt signaling pathway through suppressing expression of PIK3CA, IL2, FGF2, and VEGFA in lung tissue.

MAPKs are evolutionary-conserved enzymes connecting cell surface receptors to critical regulatory targets within cells. MAPKs signaling cascades have wide physiologic functions in the control of gene expression, cell proliferation, and programmed cell death (Chang and Karin, 2001). MAPKs, including MAPK1/2, Jun amino-terminal kinases (JNK1/2/3), and p38 proteins (p38α/β/γ/δ), have been reported to be involved in ALI/ARDS (Ying et al., 2015; Yao et al., 2017; Zhang et al., 2018). IL-1β (encoded by IL1B) and TNF-α (encoded by TNF) can activate the HIF-1 signaling pathway via MAPK cascades and PI3K, which exacerbates the inflammatory response and lung damage (Lin et al., 2019). Furthermore, proinflammatory cytokines can induce VEGF expression directly through MAPK1/2- and p38-dependent pathways (Lin et al., 2019). FGF2 can also activate MAPK1/2 via the Ras signaling pathway in lung injury (Kanodia et al., 2014). We detected four MAPK signaling pathway-associated targets (MAPK1, FGF2, IL1B, and TNF) in LPS-induced mice using Western blotting or ELISA. Our results showed that expressions of phospho-MAPK1, FGF2, IL1B, and TNF were decreased obviously, indicating that the MAPK signaling cascade was blocked effectively by ginseng.

Based on analyses of the compound–target-pathway network, the six targets (STAT3, VEGFA, FGF2, PIK3CA, MAPK1, and IL2) were the critical hubs in pathway regulation of the components in ginseng. Thus, we explored the interactions between the six targets and the components by molecular docking. As described previously (Huang et al., 2020; Yu et al., 2020), the affinity of a ligand bound to a receptor less than −5 kcal/mol denotes good binding activity. Our results showed that most of the ingredients in ginseng could combine well with STAT3, VEGFA, FGF2, PIK3CA, MAPK1, and IL2 and that hydrogen bonding and was the main form of interaction.

**FIGURE 11:**
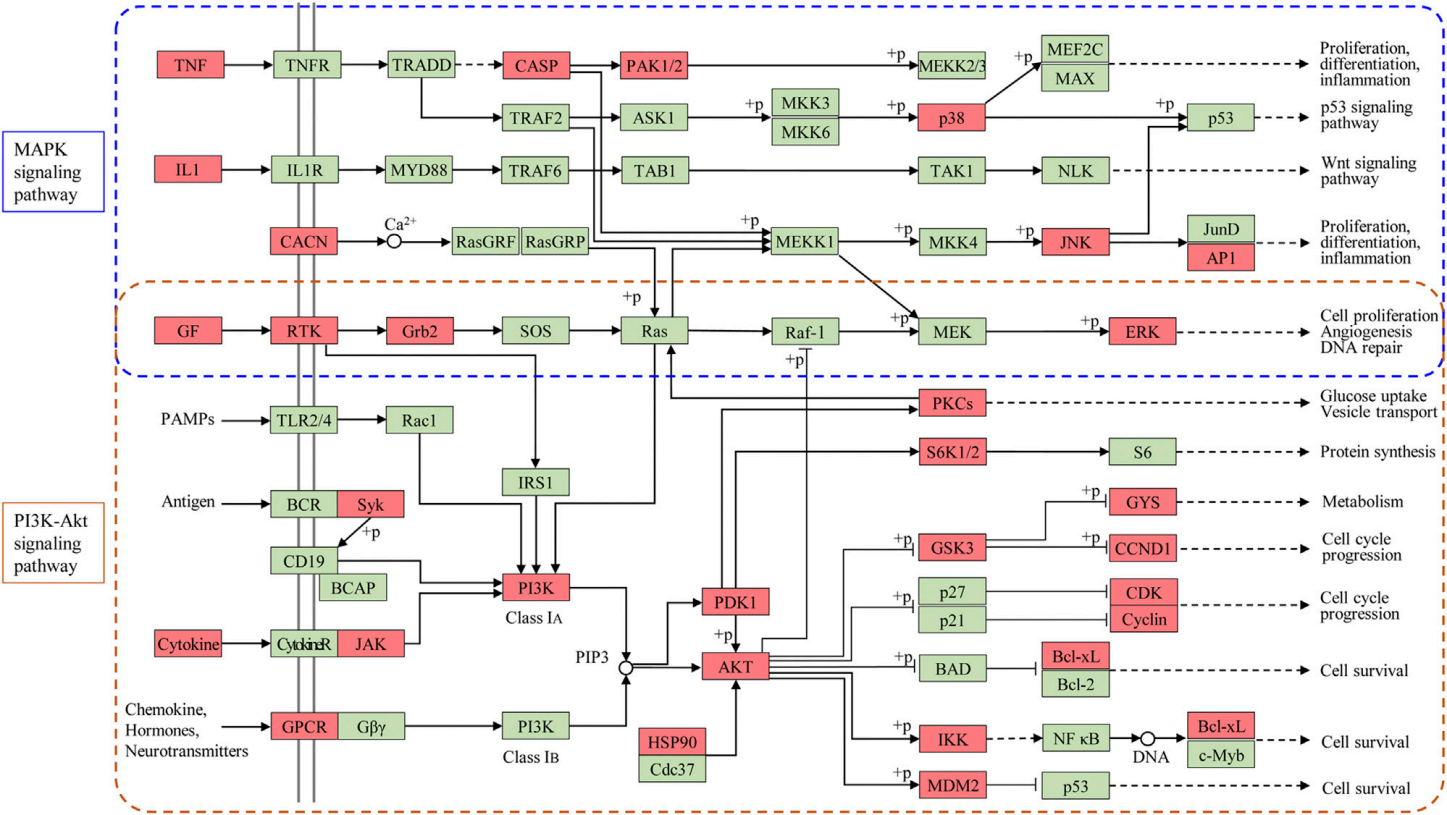
Modulating the PI3K-Akt signaling pathway and MAPK signaling pathway of ginseng against ALI/ARDS. Targets of ginseng-ALI/ARDS are colored in red, and other protein targets in the pathway are colored in green.

According to the results of network pharmacology and molecular docking, the therapeutic effect and underlying MoA of ginseng against ALI/ARDS could be summarized. That is, ginseng could alleviate the inflammatory response and pulmonary edema partly by regulation of expression of the PI3K-Akt signaling pathway and MAPK signaling pathway (Figure 11). The PI3K-Akt signaling pathway and MAPK signaling pathway were identified as the critical pathways by applying network pharmacology. In vivo experiments verified that ginseng suppressed expression of PI3K-Akt signaling pathway-associated targets (VEGFA, FGF2, PIK3CA, MAPK1, and IL2) and MAPK signaling pathway-associated targets (MAPK1, FGF2, IL1B, and TNF). The JAK-STAT signaling pathway was also enriched according to network pharmacology. Activation of the JAK-STAT signaling pathway has been reported to promote the immune response in the lung and resistance of neutrophils to apoptosis (Vier et al., 2016; Jin et al., 2018), and that STAT3 participates in regulation of the PI3K-Akt signaling pathway and MAPK signaling pathway (Bode et al., 2012; Zhao et al., 2016). We observed that p-STAT3 expression was increased in the LPS group, but ginseng could reverse this increase, indicating that ginseng reduced the number of neutrophils by inhibiting the JAK-STAT signaling pathway. To sum up, unlike only focusing on the mechanism of a single target-oriented pathway, the main active components of ginseng together with their potential therapeutic targets formed a complex network with multiple pathways. PI3K-Akt signaling pathway and MAPK signaling pathway, which were the principal pathways in the network, could interact with inflammation, immune response, and metabolism-related pathways or targets (Figure 11). Ginseng could regulate various biological processes via inhibiting the PI3K-Akt signaling pathway and MAPK signaling pathway and ultimately treat ALI/ARDS.

## Conclusion

Network pharmacology and molecular docking revealed that the PI3K-Akt signaling pathway and MAPK signaling pathway were the principal pathways of ginseng against ALI/ARDS. In vivo experiments showed that ginseng could reduce the pathologic damage, neutrophil aggregation, infiltration of proinflammatory factors, and pulmonary edema induced by LPS and inhibit the PI3K-Akt signaling pathway and MAPK signaling pathway through downregulation of expressions of STAT3, VEGFA, FGF2, PIK3CA, MAPK1, and IL2. Our study provides a theoretical basis for the beneficial effects of ginseng in the clinical treatment of patients with ALI/ARDS. Further analyses and experiments will be undertaken to confirm the association between ginseng and other pathways in ALI/ARDS.

## Data Availability

The original contributions presented in the study are included in the article/Supplementary Material, further inquiries can be directed to the corresponding author.
